# Macrophage Immunomodulation and Suppression of Bacterial Growth by Polydimethylsiloxane Surface-Interrupted Microlines’ Topography Targeting Breast Implant Applications

**DOI:** 10.3390/polym16213046

**Published:** 2024-10-29

**Authors:** Andreea Mariana Negrescu, Simona Nistorescu, Anca Florina Bonciu, Laurentiu Rusen, Luminita Nicoleta Dumitrescu, Iuliana Urzica, Anisoara Cimpean, Valentina Dinca

**Affiliations:** 1 Department of Biochemistry and Molecular Biology, Faculty of Biology, University of Bucharest, 91-95 Spl. Independentei, 050095 Bucharest, Romania; andreea-mariana.negrescu@bio.unibuc.ro (A.M.N.); simona.stroescu@inflpr.ro (S.N.); 2 Research Institute of the University of Bucharest (ICUB), University of Bucharest, 050657 Bucharest, Romania; 3 National Institute for Lasers, Plasma, and Radiation Physics, 409 Atomistilor Street, 077125 Bucharest, Romania; anca.bonciu@inflpr.ro (A.F.B.); laurentiu.rusen@inflpr.ro (L.R.); nicoleta.dumitrescu@inflpr.ro (L.N.D.); iuliana.iordache@inflpr.ro (I.U.)

**Keywords:** PDMS implants, macrophages, FBR, biofilm formation, breast implant applications

## Abstract

Since breast cancer is one of the most common forms of cancer in women, silicone mammary implants have been extensively employed in numerous breast reconstruction procedures. However, despite the crucial role they play, their interaction with the host’s immune system and microbiome is poorly understood. Considering this, the present work investigates the immunomodulatory and bacterial mitigation potential of six textured surfaces, based on linear step-like features with various regular and irregular multiscaled arrangements, in comparison to a flat PDMS surface. We hypothesise that the chosen surface geometries are capable of modulating the cellular response through mechanical interdigitation within the multiscaled surface morphology, independent of the surface chemical properties. Each type of sample was characterised from a physico-chemical and biological points of view and by comparison to the flat PDMS surface. The overall results proved that the presence of linear multiscaled step-like features on the PDMS surface influenced both the surface’s characteristics (e.g., surface energy, wettability, and roughness parameters), as well as the cellular response. Thus, the biological evaluation revealed that, to different degrees, biomaterial-induced macrophage activation can be mitigated by the newly designed microtextured surfaces. Moreover, the reduction in bacteria adherence up to 90%, suggested that the topographical altered surfaces are capable of suppressing bacterial colonisation, therefore demonstrating that in a surgical environment at risk of bacterial contamination, they can be better tolerated.

## 1. Introduction

With the continuous evolution of the biomedical research and surgical intervention field, numerous implantable biomedical devices are intensively studied and employed with the purpose of improving the patient’s life quality through the maximisation of outcomes and minimization of after-surgery complications [1]. Following their first introduction to the market, breast implants have also gone through a process of evolution, from an early history full of controversies to a multi-million-dollar industry [1,2] with over 320,000 breast implant-based reconstruction and augmentation surgeries performed annually in the USA alone [3].

Polydimethylsiloxane (PDMS) is an extremely versatile polymer with a wide range of applicability, mainly due to its favourable characteristics, such as high oxygen permeability, chemical stability, tuneable stiffness and very low surface tension [4]. More importantly, PDMS-based biomaterials possess an excellent biocompatibility, as reported by numerous in vitro and in vivo studies on different cell types, such as bone cells [5,6], skin cells [7,8,9,10], inflammatory cells [11,12] etc., and a high resistance to biodegradation, thus highlighting their potential as suitable candidates for various biomedical applications. For example, silicone is used in the human body with a high level of clinical success in the form of urinary stents and catheters [13], central venous catheters stents [14], peacemakers [15], artificial skin [16], and numerous medical/surgical implants [16]. For catheters or drains, the silicone’s transparent, flexible, inert, biocompatible, and lubricating nature makes it ideal for tubing, while its high oxygen permeability and biocompatibility meet the criteria required for an adequate wound dressing [16]. In addition, in its gel form, silicone is used to treat hypertrophic burn scars, whereas silicone rubber is utilised in the fabrication of prostheses [16]. In this context, due to its superior properties and high usability, PDMS became the standard polymeric material used in breast implant fabrication. However, despite its advantages, the PDMS-based implant can sometimes elicit undesirable and unforeseeable chronic immune responses which, in turn, will lead to an excessive foreign body response (FBR) in patients [17]. This excessive immune-driven response is accountable for one of the most common complications following breast implant surgery, namely the formation of a fibrotic capsule around the implantable device (i.e., capsular contracture) [18]. While the typical evolution of FBR results in the development of an initial soft, but slightly firm, functional collagenous capsule, which does not affect the appearance or feel of the breast and helps maintain the implant in position [19], in a subset of individuals, the immune response is amplified, and the tissue becomes abnormally thicker, denser, and less pliable [20], leading to chronic pain, breast firmness, and an unwanted position, coupled with a poor aesthetic look, which more than often requires corrective surgical intervention [18].

Since the capsular contracture is initiated once the cells and proteins have come into contact with the PDMS substrate, it was suggested that the surface texture of the implant plays a significant impact on its development [21]; therefore, in recent decades, various topographical surface alteration approaches have been utilised in order to diminish the chronic immune response induced by the biomaterial and implicitly minimise the occurrence of this phenomenon [22]. According to data reported in the literature, micro- and nanometre scaled topographical characteristics can effectively alleviate the fibrotic process, and implicitly implant failure, through local immune response modulation [23]. In this light, in the current study, six new textured surfaces, based on lines step-like features with various regular and irregular multiscaled arrangements, have been specially designed with the purpose of evoking a diminished inflammatory response and a suppressed bacterial adhesion.

Thus, in order to determine the ability of these microtopographically altered PDMS surfaces to mediate the inflammatory response and FBR in an in vitro microenvironment, macrophages—essential inflammatory cells involved in important in vivo processes such as tissue repair, remodelling and wound healing [24]—were cultured on these surfaces. With this in mind, the in vitro behaviour of RAW 264.7 macrophage-like cells, grown in contact with the modified PDMS surfaces, was investigated in terms of cell viability/proliferation, morphology, inflammatory mediators’ secretion, and potential to sustain their fusion into multinucleated foreign body giant cells (FBGCs). Furthermore, beside the immunobiological risk, data reported in the literature indicate that the presence of bacteria at the implantation site could represent a potential factor in the development of the capsular contracture. Similar to other implantable devices, breast implants are also susceptible to bacterial colonisation and biofilm formation [25], and since the proposed geometries are considered bacteria-sized, therefore acting like traps for bacterial microorganisms, in the present study, the adhesion of *Staphylococcus aureus* (*S. aureus*) on flat and textured PDMS was also evaluated.

## 2. Materials and Methods

### 2.1. Microtextured PDMS Substrate Obtaining and Characterisation

#### 2.1.1. Microtextured PDMS Replication

A total of 35 g of Dow Sylgard 184 base and curing agent (from The Dow Chemical Co., Midland, MI, USA) were mixed in 10 to 1 ratio, poured on top of the moulds obtained by photolithography (Digital Optics Corp., Charlotte NC, USA), and cured at room temperature for 48 h. The resulting PDMS samples were subsequently removed from the mould and the aspect ratio was maintained without notable changes (Figure S1), immersed for 30 min in ethanol, washed in 3 steps by sonication in double distilled water (10 min cleaning step), and dried with Argon gas before Ozone lamp treatment. A total of 250 nm multiscaled step-like structures of various micron-sized lengths and spacings, as well as different distributions, were chosen for this study. The samples were named according to the linear pattern distribution within the replicated areas as follows: overlapped linear pattern (OLP); mixed sequential parallel step-like pattern (SPSP); overlapped irregular linear pattern (OILP); and confluent step-like linear pattern (CSLP). Moreover, the following two regular linear patterns were proposed as alternatives to the irregular patterns: gradient circular linear pattern (CLP) and parallel linear pattern (PLP). Flat PDMS was used as the control for all of the experiments.

#### 2.1.2. Substrates Characteristics

The morphological characteristics of the PDMS-microtextured substrates were evaluated using atomic force microscopy (AFM) and scanning electron microscopy (SEM-JSM-531 Inspect S Microscope from FEI Company, Hillsboro, OR, USA) (accelerating voltages: 5–25 kV). A total of 10 nm Au (Agar Scientific Ltd., Essex, UK) were sputtered on all the samples prior to SEM analysis. The samples containing grown mammalian cells were washed with PBS and fixed for 20 min with 2.5% glutaraldehyde solution prepared in PBS. Then, the samples were gradually dehydrated with 70%, 90%, and 100% ethanol solutions for 15 min, two exchanges for each step followed by two rounds (3 min each) of incubation with 50%, 75%, and 100% hexamethyldisilazane (HDMS, Sigma-Aldrich, St. Louis, MO, USA) ethanol solution. Samples were air-dried overnight in a chemical fume hood and metalized prior to scanning.

Detailed profiles and the structures’ walls were obtained in non-contact mode by AFM investigations (XE100 AFM, Park Systems, Suwon, South Korea). Surface topography parameters measurements (amplitude parameters: Sa (µm) = arithmetical mean height; Sq (µm) = root mean square height; St (µm) = peak to valley height; Sz = maximum peak to valley distance; Sv = maximum valley depth; Sp = maximum peak height; Ssk = skewness; excess Sku = kurtosis) were performed using a XP-2 Stylus-Profiler System (Advanced Stylus Profiling System Version 5.5.5) (1.5 Å vertical resolution, applied force of 2 mg, and a scanning rate of 0.01 mm/s), while Ambios Technology and software package TrueMap v4. Ambios Technology were used for the surface roughness parameters. The results are presented as the mean ± standard deviation (SD) for 60 measurements. Data are displayed as the mean of at least six 140 × 140 μm^2^ areas of each substrate surface and ± indicates standard deviation.

#### 2.1.3. Characterisation of Wettability Through Contact Angle (CA) Measurements and Surface Energy Analysis

The clean PDMS samples were exposed for 2 h to an UV Ozone lamp. The water contact angles on the PDMS surfaces were assessed using a KSV CAM101 microscope (KSV Instruments Ltd., Espoo, Finland) by employing the conventional sessile drop technique at a temperature approximately equal to the room temperature (around 20 °C). Ultrapure water and di-iodomethane sourced from Sigma Aldrich, St. Louis, MO, USA, were employed in order to determine the contact angles, facilitating the calculation of surface free energy (SFE) via the Owens, Wendt, Rabel, and Kaelble (OWRK) approach.

### 2.2. In Vitro Biological Examination

In order to maintain their hydrophilic character for an extended period, compared to when they are exposed to air, as well as to shield their surface from atmospheric contaminants, which further prolong the hydrophilic state, all the PDMS samples were exposed for 2 h to an UV Ozone lamp, placed in sterile distilled water and kept at 4 °C until their use.

#### 2.2.1. Evaluation of the Antimicrobial Potential

*S. aureus*, one of the main microorganisms responsible for breast surgery-related infections, was used as the control bacterium in this study. Following sterilisation, 600 µL of *S. aureus* (American Type Culture Collection 25923) suspensions were placed on top of the samples and incubated at 37 °C, in a humidified atmosphere with 5% CO_2_, for various time periods, from 24 h up to 25 days. After each period of incubation, SEM microscopy was employed to visualise the subsequent biofilm development on the textured or smooth PDMS surfaces. For the SEM analysis, after incubation, the samples were subjected to three consecutively washes with PBS and 1 mL methanol was added. After 15 min, the methanol was removed, and the samples were dried and observed.

#### 2.2.2. Evaluation of the Cellular Behaviour

##### Cell Culture Model

Commercially available murine macrophages RAW 264.7 cells (American Type Culture Collection, Manassas, VA, USA) were seeded directly onto the surface of the sterile samples at different initial cell densities, in accordance with the investigations performed. Thus, to investigate the cell survival, proliferation, and morphology, the initial density was 10^4^ cells/cm^2^, while for the assessment of the inflammatory mediators’ release and FBGCs formation, the used densities were 8 × 10^4^ cells/cm^2^ and 5 × 10^4^ cells/cm^2^, respectively. The macrophages were multiplied in Dulbecco’s Minimal Essential Medium (DMEM, Sigma-Aldrich Co., St. Louis, MO, USA), supplemented with 10 vol. % foetal bovine serum (Gibco, Life Technologies Corporation, Grand Island, NY, USA) and 1 vol. % penicillin/streptomycin (10,000 units/mL penicillin to 10 mg/mL streptomycin) (Sigma-Aldrich Co., St. Louis, MO, USA) at 37 °C in a humidified atmosphere of 5% CO_2_. The culture medium was refreshed every two days until their seeding onto the PDMS-based substrates. Moreover, the in vitro studies were performed both in standard culture and pro-inflammatory (stimulation with 100 ng mL^−1^ lipopolysaccharide (LPS) from *Escherichia coli*) conditions.

##### The Assessment of Cellular Viability and Proliferation

The cellular viability/proliferation of the RAW 264.7 cells was quantitatively evaluated at 1 and 3 days post seeding by employing Cell Counting Kit (CCK)-8 (Sigma Aldrich Co., St. Louis, MO, USA), as reported [26]. In the end, a microplate reader was used to determine the optical density (OD) of the reaction product at 450 nm (FlexStation 3 microplate reader, Molecular Devices, Sai Jose, CA, USA).

##### Fluorescent Labelling of the Actin Cytoskeleton Organisation

By labelling the F-actin fibres with phalloidin coupled with Alexa Flour 488 (protocol described in a previously study) [26], the macrophages’ cytoskeleton organisation was evaluated at 1 and 3 days post seeding. The stained cells were observed with a fluorescence microscope (Olympus IX71, Olympus, Tokyo, Japan), while the microscopic fields were captured using the cellSense Dimension acquisition system (Version 4.1). Furthermore, the spreading area and roundness of the RAW 264.7 cells (30 individual cells/condition) were quantified using the Image J software (Version 1.53c, National Institutes of Health, Bethesda, MD, USA). By definition, roundness, with value between 0 (highly elongated) and 1 (perfectly round), was calculated using the following formula: (4π × (cell area)/(cell perimeter)^2^).

##### Quantification of the Inflammatory Mediators

The inflammatory activity of the RAW 264.7 cells grown onto the developed PDMS surfaces was investigated by quantifying the concentration levels of the tumour necrosis factor (TNF)-α, interleukin (IL)-1β, and IL-10 accumulated in the cell culture medium after 2 days of culture, with the help of specific sandwich enzyme-linked immunosorbent assay (ELISA) kits, as described in the accompanying instructions leaflet (R&D Systems, Minneapolis, MN, USA). The ODs of the final products were determined using a microplate reader (FlexStation 3 microplate reader, Molecular Devices, San Jose, CA, USA) and their corresponding concentrations, expressed in pg/mL, were calculated by reporting to their standard curve. In addition, the nitric oxide (NO) concentration was also analysed using the Griess reagent (Promega, Madison, WI, USA), as we previously reported [27]. The product was then measured spectrophotometrically at a wavelength of 550 nm using a microplate reader (FlexStation 3, microplate reader, Molecular Devices, San Jose, CA, USA), and the resulting nitrite concentration was calculated from a sodium nitrite standard curve through extrapolation.

##### Foreign Body Giant Cells’ Formation Assessment

To determine whether the analysed surfaces have the potential to induce the formation of FBGCs, the macrophages were cultured under pro-inflammatory conditions (treatment with 100 ng mL^−1^LPS) for one week, with medium changing every 2 days. At the end of the experimental time point, the samples were subjected to the protocol described in [27], and the labelled cells were visualised with an Olympus IX71 microscope (Olympus, Tokyo, Japan). The microscopic images were captured with the cellSense Dimension acquisition system (Version 4.1). Furthermore, the multinuclear formation of FBGCs was also investigated through a quantitative method—establishment of the “multinuclear index”, which was calculated as the percentage of nuclei in multinuclear cells with ≥3 nuclei against the total number of nuclei.

### 2.3. Statistical Analysis

GraphPad Prism software (Version 6, GraphPad, San Diego, CA, USA) was used to perform the statistical analysis (one-way/two-way ANOVA (Turkey’s/Bonferroni’s multiple comparison) tests). The values are expressed as means ± SD and *p* values of <0.05 were considered to be statistically significant.

## 3. Results

### 3.1. Surface Characteristics

From the data reported in the literature, it became apparent that the texture of a breast implant surface can play a significant role in the development of anaplastic large cell lymphoma, a phenomenon associated with bacterial colonisation, infection, and a prolonged pro-inflammatory state [28]. Therefore, when designing breast prostheses with topographic features, special attention must be paid to the appropriate surface characteristics that can hinder the attachment of bacteria and can modulate the macrophages’ activity towards an attenuate inflammatory response. Therefore, the proposed topographies considered the dimensions, arrangement, and intended functional outcomes related to bacterial adhesion and macrophage interaction, consisting of a rectangular step-like feature and a 250 nm step size, with multiscaled in depth distribution as well spacing varying from 900 nm to tens of microns.

**Figure 1:**
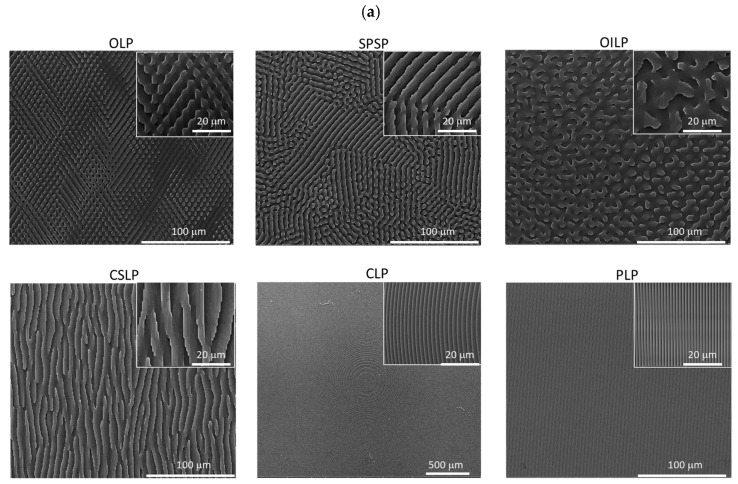

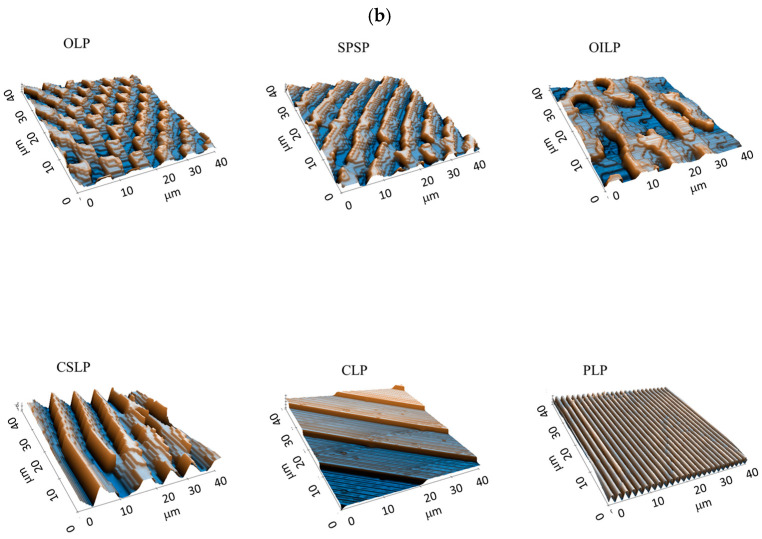
SEM (**a**) and AFM (**b**) images of PDMS replicated surfaces depicting step-like structures and the different arrangements on the surface.

The SEM (Figure 1a) and AFM (Figure 1b) images reveal the detailed microscale features of the interfaces replicated in PDMS. The new textured PDMS surfaces maintained a regular linear step-like feature in an overlapping design with 2 to 3 microns spacing between top structures and a maximum depth of 2 microns (i.e., Overlapped Linear Pattern—OLP) or a mixture of overlapped and parallel pattern domains (i.e., sequential parallel step-like pattern design—SPSP), which seeks to understand how mixed textures might offer superior resistance towards bacterial colonisation while fostering an anti-inflammatory microenvironment.

As an alternative to a regular features arrangement, a combination of overlapped irregular/interrupted micron-sized length linear patterns (10 to 50 micrometres, with varying spacing) that overlap randomly across the surface features (OILP), as well as another one consisting of continuous, step-like linear features that merge, creating barriers (CSLP), were also proposed with the aim of creating a complex surface texture capable of disrupting bacterial adherence by grouping the features in a trap-like manner.

Two more types of strictly regular linear patterns were proposed as alternatives to the irregular or combined regular and irregular ones: a Circular Linear Pattern—CLP—that includes circular lines with varying gradients, which could influence the bacterial distribution and macrophage movement in a unique manner; and a Parallel Linear Pattern—PLP—comprising straight, parallel lines, evenly spaced across the surface with line widths of 900 nm up to 1 micrometre, spaced 1 micrometre apart.

When compared to the previously reported textured implant surfaces (Sa = 8.24 μm; Sz = 40 μm), the values for all of the topographically modified surface areas were characterised by a decreased roughness (Sa below 5 μm), with the exception of the CLP that had an Sa = 10.77 μm. As shown by the AFM and SEM images (Figure 1a,b), the textured surfaces were characterised by a step-like type profile, containing microscale features as well as depth step features of 250 nm, culminating in a maximum depth of 1 μm for all the samples, while the lateral step features length and width varied according to Table 1. Moreover, as the analysed surfaces mainly revealed peaks with flat profiles as well as valleys, the calculated skewness (Sku) and kurtosis (Ku) showed positive values, indicating an excess in skewness and kurtosis for all the samples except OLP, for which a negative skewness characteristic to a valley-predominant profile was obtained (Table 1). This indicates, that in general, the mean and median were greater than the mode, and that the OLP surface presented more valleys than peaks. When compared to the analysed smooth silicone implant surface, the values followed the trend for positive skewness and excess kurtosis values (Ssk: 0.027and SKU: 1.833).

**Figure 2:**
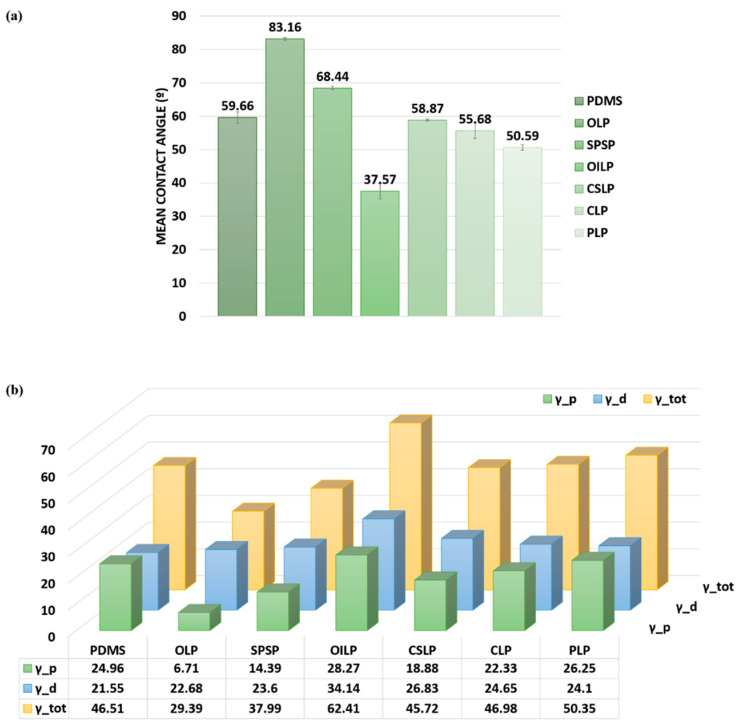
(**a**) Modifications in surface wettability evaluated by contact angle measurements of PDMS surfaces; (**b**) Surface free energy components.

When untreated due to the presence of the −CH_3_ groups [29,30], the PDMS surface showed hydrophobic properties with a WCA of 104°, which frequently restricts its use in solutions containing biological samples [31,32]. Therefore, the PDMS surfaces were exposed to UV Ozone and the values were mainly moderately hydrophilic, exhibiting intermediate WCA from 50° to 83° for the OLP, PLP, CLP, SPSP, and CSLP topographies, whereas the OILP substrate displayed the lowest contact angle (37.57°), indicating a more hydrophilic surface (Figure 2). This significant difference can be explained by the exposed area given by the height distribution of the steps and their grouping on the surface, which gave the smallest roughness values when measured by the optical profilometer (Table 1).

In general, this type of treatment of the PDMS surfaces led to the formation of polar groups (-OH, -COOH) on the surface, a phenomenon which can explain the decrease in the contact angle of the untreated surfaces (values above 103°), as well as the increase in both total surface energy and its polar component. Moreover, as the formation of a thin silica-like layer on the surface contributes significantly to the hydrophilic properties of UV Ozone-treated PDMS, in order to decrease its degradation over the testing time, the samples were kept in aqueous media.

### 3.2. In Vitro Biological Behaviour

#### 3.2.1. The Antimicrobial Potential

**Figure 3:**
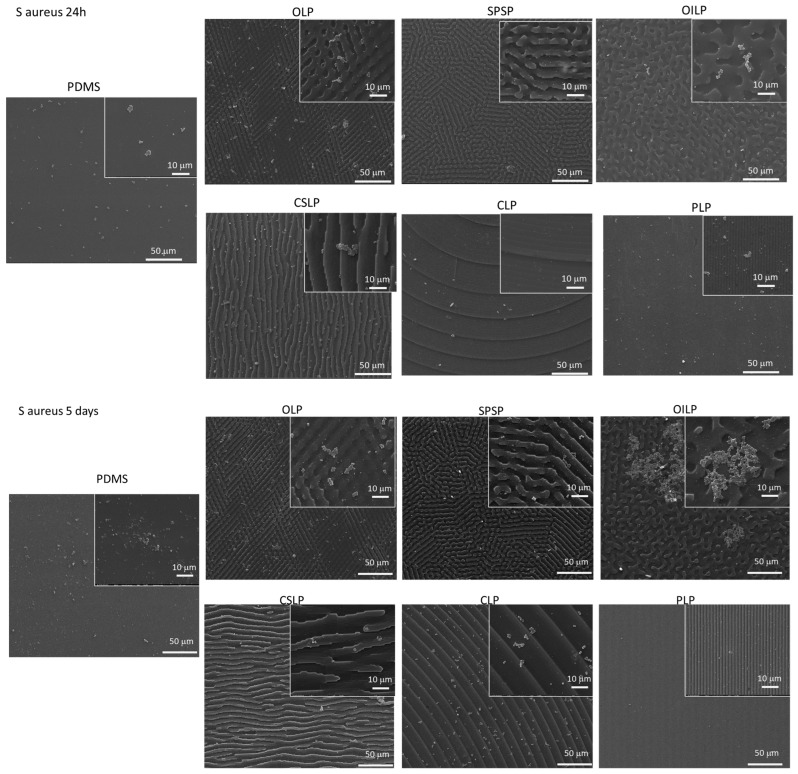

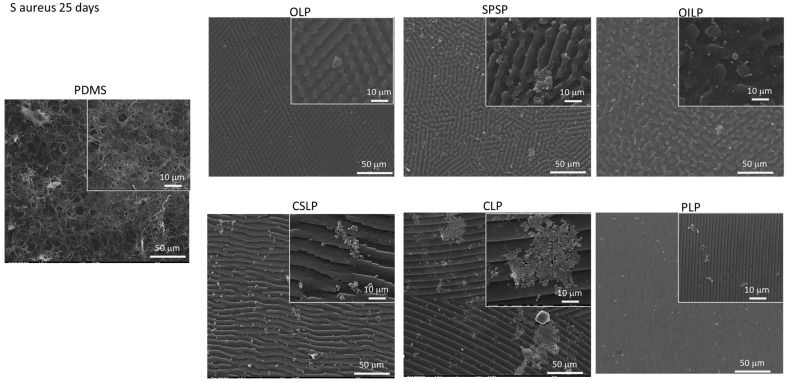
SEM images of *S. aureus* biofilm formation at 1, 5, and 25 days onto the flat and structured PDMS surfaces, respectively.

As it becomes exceedingly challenging to eradicate bacteria or mechanically eliminate biofilms from solid surfaces, one potential strategy is designing a specific surface topography capable of hindering bacterial adhesion through hydrodynamics. For example, in their study, Epstein et al. [33] were able to prevent *Pseudomonas aeruginosa*, *S. aureus*, and *E. coli* adhesion over 7 days on slippery liquid-infused porous surfaces. Another promising design was proposed by Chien et al. [34], where a sharkskin-like topography led to a significant alteration in bacterial attachment and biofilm formation by providing specific height profiles. In a similar manner, the interrupted lines’ type of topography with various arrangement of features proved their efficiency in hindering *S. Aureus* adhesion not only for the first 24 h but also after 25 days (with a max. of 5–10% surface coverage), excepting the circular gradient like pattern (CLP), where the accumulation of bacteria colonies covered more than 60% of the surface (Figure 3).

This aspect can be explained through the capacity of the textured PDMS surfaces to generate air-water interfacial regions that were previously inaccessible for the bacterial attachment of *S. aureus*. This illustrates the potential of the proposed microstructures to influence the initial stages of bacterial attachment transiently. In addition to chemical sensing, bacteria cells are also capable of sensing surface-associated mechanical cues [35]. The reason is that when the microscale features of the surface are in the same order as bacteria, their initial attachment is influenced by cell membrane deformation and physico-chemical forces [35]. When the interrupted and/or mixed linear features surfaces were analysed, our results were corroborated by those of Linklater et al. [36] on linearly increasing heights surface geometries in black silicon, where it was demonstrated that the greatest bactericidal effects against *S. aureus* bacteria were exhibited by the smaller, densely packed pillars. Moreover, the observation on the engineered gradient linear pattern surfaces (CLP), characterised by well-defined step feature dimensions of at least five orders of magnitude larger than a single bacterium, demonstrated an enhanced bacterial attachment based on the highest provided available contact area, and therefore, the ability to shelter *S. aureus*, which is in accordance with the effects described by Helbig et al. [37]. It is noteworthy that bacterial attachment was markedly enhanced on the smooth substrate over time, whereas the protruding surface features impeded further biofilm development. There are studies underlining the role of surfaces with a feature smaller than a single microorganism in reducing bacterial adhesion [37,38,39,40,41], including biomimetic topographies (i.e., lotuses [42], cicadae [43], sharks [34], geckos [44,45], and dragonfly wings [46]), which are however linked to contaminant-free surfaces and superhydrophobic physical surface structures (combined with specific compounds onto their surfaces).

#### 3.2.2. RAW 264.7 Macrophages’ Viability/Proliferation

**Figure 4:**
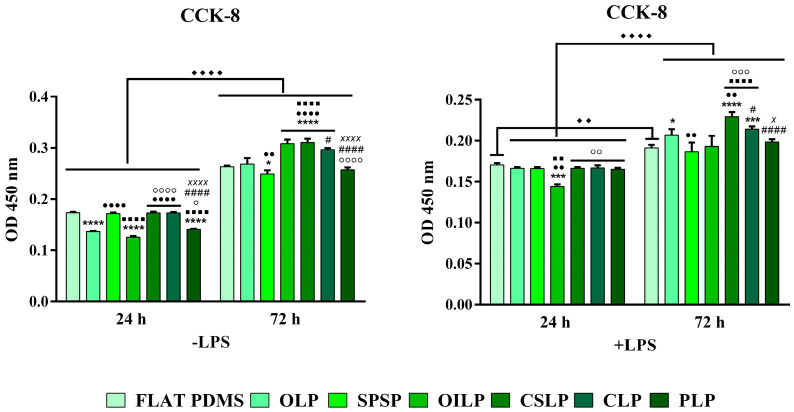
The viability/proliferation rate assessed by the CCK-8 assay after 1 and 3 days, under both standard (-LPS) and pro-inflammatory (+LPS) conditions (n = 3, mean ± SD, **** *p* < 0.0001, *** *p* < 0.001, * *p* < 0.05 vs. FLAT PDMS; ●●●● *p* < 0.0001, ●● *p* < 0.01 vs. OLP; ■■■■ *p* < 0.0001, ■■ *p* < 0.01 vs. SPSP; ○○○○ *p* < 0.0001, ○○○ *p* < 0.001, ○○ *p* < 0.01, ○ *p* < 0.05 vs. OILP; #### *p* < 0.0001, # *p* < 0.05 vs. CSLP; xxxx *p* < 0.0001, x *p* < 0.05 vs. CLP). The significance level between the two groups (24 h vs. 72 h) was ♦♦♦♦ *p* < 0.0001 (-LPS) and ♦♦♦♦ *p* < 0.0001, ♦♦ *p* < 0.01 (+LPS).

Since surface architecture alone, independent of the chemical and mechanical features of the biomaterial itself, can regulate important biological processes at the cell-implant interface [47] in the first step, a CCK-8 assay was used to determine how microtextured surfaces can influence the survival and proliferation rates of the RAW 264.7 macrophages. Figure 4 shows that after 1 day of culture, the number of viable metabolically active cells cultured onto the surface of the tested supports were similar to that exhibited on the smooth surface, with the exception of the OILP, OLP and PLP samples, where an average decrease of 22.6% in the recorded OD values could be observed, but only for the cells not previously treated with LPS (-LPS). However, at 3 days post seeding, the cell proliferation rates differed significantly between the analysed surfaces, with an increase in the number of macrophages seeded directly on to the OILP, CLP and CSLP substrates, the trend observed in both standard (average increase of 24.8%) and pro-inflammatory (average increase of 10.5%) culture conditions. Furthermore, it has also been revealed that the prolonged exposure of macrophages to the pro-inflammatory stimulus resulted in a significant reduction in their metabolic activity, with an average decrease of 38.4%, in comparison to the unstimulated cells. This phenomenon is widely reported in the literature and is mainly attributed to the inhibitory effect of the bacterial endotoxin on the cells’ proliferative potential [48]. Altogether, these results confirm the time-dependent growth-supporting capacity of all investigated microtextured surfaces, regardless of the culture conditions (*p* < 0.0001). This can be correlated to previous studies on other types of microtextured silicone-based implants fabricated using electrospun fibres as a sacrificial template [23], which demonstrated the employment of various microtopographic features on PDMS surfaces, with a favourable effect on the survival and proliferation rates of cells in the early stages of culturing.

#### 3.2.3. RAW 267.4 Cells’ Morphological Features

**Figure 5:**
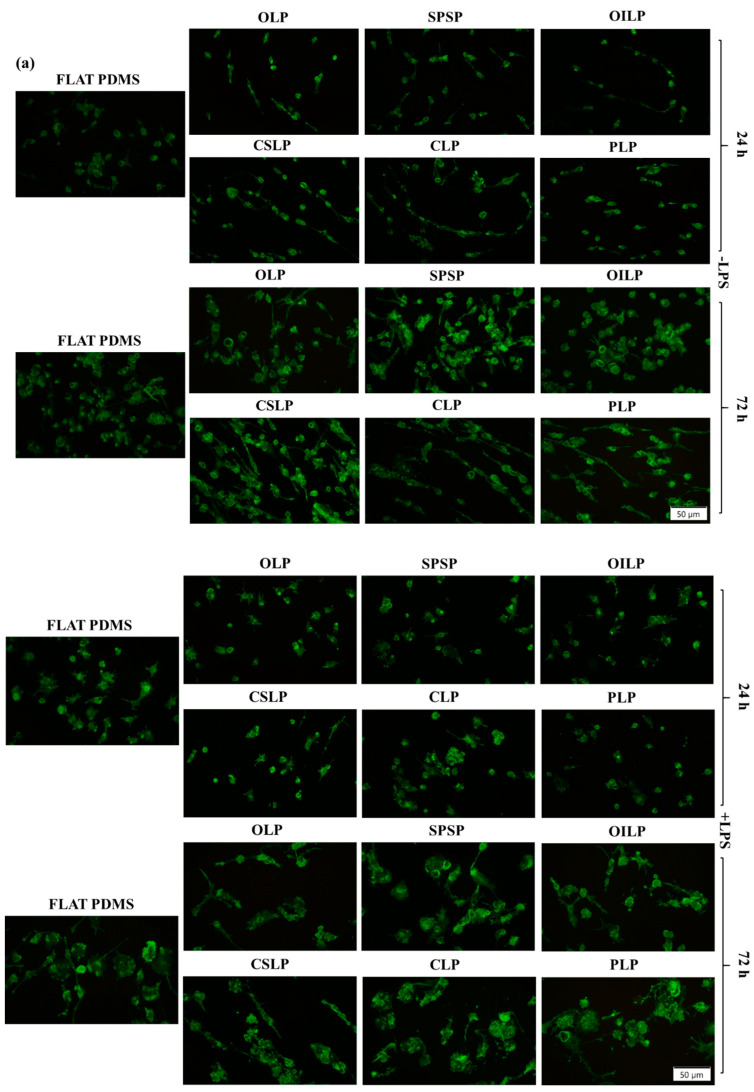

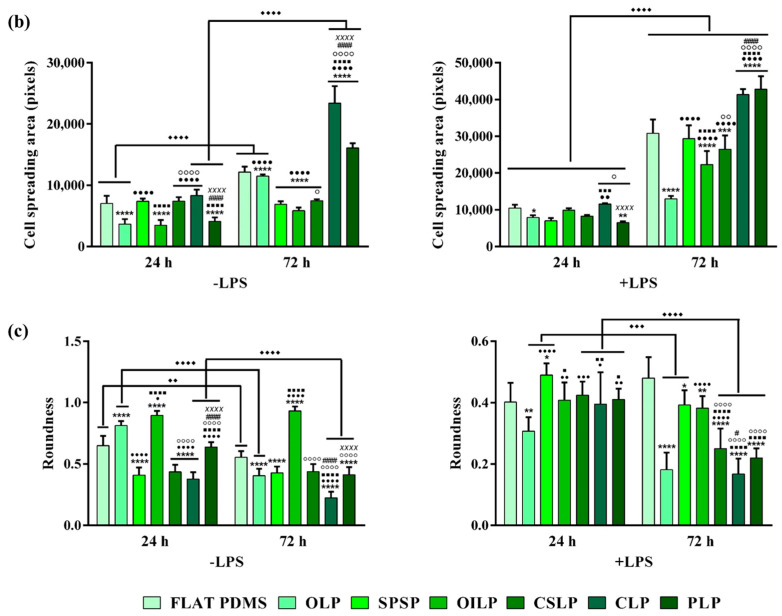
(**a**) The morphological features exhibited by the RAW 264.7 macrophages on the analysed PDMS surfaces after 1 and 3 days of culture, under standard (-LPS) and pro-inflammatory (+LPS) conditions (green fluorescence—actin cytoskeleton). Scale bar represents 50 µm; (**b**) Cell spreading area quantification (n = 30 cells, means ± SD; **** *p* < 0.0001, *** *p* < 0.001, ** *p* < 0.01, * *p* < 0.05 vs. FLAT PDMS; ●●●● *p* < 0.0001, ●● *p* < 0.01 vs. OLP; ■■■■ *p* < 0.0001, ■■■ *p* < 0.001 vs. SPSP; ○○○○ *p* < 0.0001, ○○ *p* < 0.01, ○ *p* < 0.05 vs. OILP; #### *p* < 0.0001 vs. CSLP; xxxx *p* < 0.0001 vs. CLP). The significance level between the two groups (24 h vs. 72 h) was ♦♦♦♦ *p* < 0.0001; (**c**) Cell roundness quantification (n = 30 cells, means ± SD; **** *p* < 0.0001, ** *p* < 0.01, * *p* < 0.05 vs. FLAT PDMS; ●●●● *p* < 0.0001, ●●● *p* < 0.001, ●● *p* < 0.01, ● *p* < 0.05 vs. OLP; ■■■■ *p* < 0.0001, ■■ *p* < 0.01, ■ *p* < 0.05 vs. SPSP; ○○○○ *p* < 0.0001 vs. OILP; #### *p* < 0.0001, # *p* < 0.05 vs. CSLP; xxxx *p* < 0.0001 vs. CLP). The significance level between the two groups (24 h vs. 72 h) was ♦♦♦♦ *p* < 0.0001, ♦♦ *p* < 0.01 (-LPS) and ♦♦♦♦ *p* < 0.0001, ♦♦♦ *p* < 0.001 (+LPS).

In an in vivo microenvironment, the extracellular matrix (ECM) exposes the cells to a broad variety of morphological stimuli, which in one way or another will influence their behaviour in terms of shape, adhesion, migration, etc. [49]. This phenomenon is referred to in the literature as “contact guidance” and occurs as a result of the rearrangement of the interconnected ECM fibre structures, which in turn activates cell migration and shape acquisition [50]. Similar to the natural ECM, the surface topography of an implant can also regulate the cells’ behaviour by influencing their adhesion and cytoskeleton organisation [51,52,53]. Thus, by altering the mechanosensing at the cell-implant interface, a tailored cellular behaviour could be obtained. In light of the aforementioned considerations, an investigation was conducted into the cellular adhesion and morphology of macrophages cultured on the tested surfaces. This was achieved through the use of F-actin cytoskeleton fluorescence labelling, employing Alexa Fluor 488-conjugated phalloidin. As seen in Figure 5a, the fluorescence microscopy images revealed that depending on the employment of micropatterning, the RAW 264.7 cells cultured onto the analysed surfaces exhibited different morphological characteristics. Therefore, at 1 day post seeding, in the absence of the pro-inflammatory stimulus (-LPS), the cell population found on the microtextured PDMS was heterogenous, displaying both cells with round-shaped bodies (distinctive for unstimulated macrophages), but also cells with elongated spindle-like morphologies. Moreover, this phenomenon could be better observed after 3 days of culture, where the cells grown in contact with the surface of the majority of the analysed supports, displayed elongated bodies along the alignment patterns, whereas on the smooth surface, they were randomly orientated and exhibited a predominantly cytoskeleton organisation, characteristic of normal cells. These differences in cell adhesion and morphology patterns between the microtextured and smooth surfaces are derived from the altered interactions between cells and the underlying topography characteristics [54]. In contrast, upon LPS treatment at both experimental time points, the cells lost their micropatterned alignment, exhibiting a random attachment trend coupled with an altered morphology, a high degree of spreading, and numerous filopodia. Existing data associates this morphological behaviour with a switch in the macrophage polarisation state towards an activated, migratory, and pro-inflammatory M1 phenotype [55]. Moreover, fluorescent labelling also revealed the formation of podosomes (dot-like actin structures) with important roles in adhesion and mechanosensing [56]. In addition, the morphometric semi-quantitative analysis of cell area spreading (Figure 5b) and roundness (Figure 5c) confirms the morphological findings, further demonstrating the influence of microtexturing on cellular attachment and morphological behaviour. Thus, as seen from the graphics (Figure 5b,c), after the prolonged exposure to pro-inflammatory stimulus, the majority of the adherent cells, found on the microtextured PDMS surfaces, suffered morphological alterations, adopting elongated bodies and larger cell spreading areas.

Our observations regarding the microtexturing’s ability to modulate the morphological behaviour of immune cells, are in accordance with the work of Choi et al. [23], who demonstrated how the presence of microtopographic features on PDMS surfaces can affect the distribution and cytoskeleton organisation of RAW 264.7 macrophages, leading to a directional orientation parallel to the micropatterned alignment. Similarly, Chen et al. [57] reported topography-mediated changes in the morphology of macrophages grown on an altered PDMS-microtextured surface, in comparison to a smooth substrate. Moreover, in another study, Bettinger et al. [58] demonstrated that cells grown in contact with smaller pitch-sized microstructures exhibited spindle-like morphologies, a higher disposition for alignment and an increase in the cell’s spreading area, in comparison to flat surfaces.

#### 3.2.4. The Release of Inflammatory Mediators by RAW 264.7 Cells

**Figure 6:**
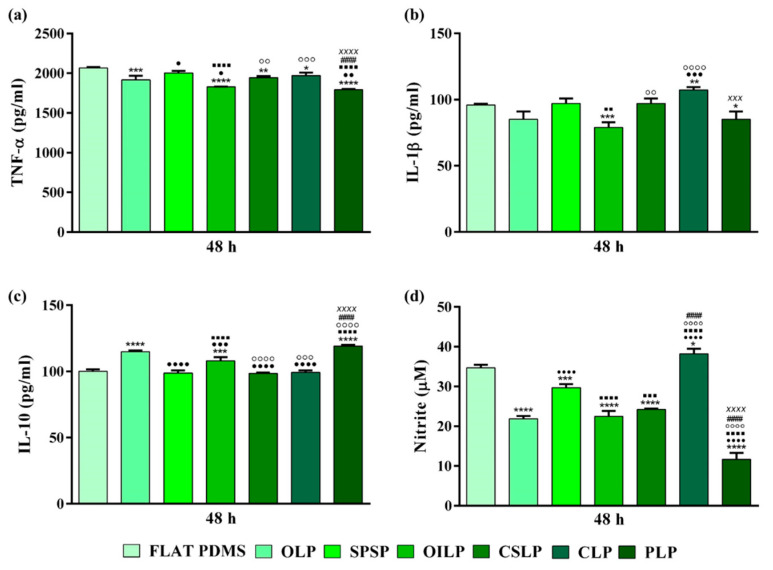
Quantification of the inflammatory mediators released after 2 days of culture (100 ng mL^−1^). ELISA measurement of: (**a**) TNF-α (**** *p* < 0.0001, *** *p* < 0.001, ** *p* < 0.01, * *p* < 0.05 vs. FLAT PDMS; •••• *p* < 0.0001, •• *p* < 0.01, *• p < 0.05* vs. OLP; ■■■■ *p* < 0.0001 vs. SPSP; ○○○ *p* < 0.001, ○○ *p* < 0.01 vs. OILP; #### *p* < 0.0001 vs. CSLP, xxxx *p* < 0.001 vs. CLP); (**b**) IL-1β (*** *p* < 0.001, ** *p* < 0.01, * *p* < 0.05 vs. FLAT PDMS; ••• *p* < 0.001 vs. OLP; ■■ *p* < 0.01 vs. SPSP; ○○○ *p* < 0.001, ○○ *p* < 0.01 vs. OILP; xxx *p* < 0.001 vs. CLP); (**c**) IL-10 (**** *p* < 0.0001, *** *p* < 0.001 vs. FLAT PDMS; •••• *p* < 0.0001, ••• *p* < 0.001 vs. OLP; ■■■■ *p* < 0.0001 vs. SPSP; ○○○○ *p* < 0.0001, ○○○ *p* < 0.001 vs. OILP; #### *p* < 0.0001 vs. CSLP; xxxx *p* < 0.0001 vs. CLP); (**d**) Extracellular NO measurement by the Griess reagent (**** *p* < 0.0001, *** *p* < 0.001, * *p* < 0.05 vs. FLAT PDMS; •••• *p* < 0.0001 vs. OLP; ■■■■ *p* < 0.0001, ■■■ *p* < 0.001 vs. SPSP; ○○○○ *p* < 0.0001 vs. OILP; #### *p* < 0.0001 vs. CSLP, xxxx *p* < 0.0001 vs. CLP). Results are expressed as means ± SD (n = 3).

It is a well-known fact that a fully functional healing process that results in a healthy new tissue, requires the satisfactory resolution of inflammation, or else the acute inflammatory phase will turn into chronic inflammation, a prolonged inflammatory state accompanied by macrophage fusion, formation of FBGCs, excessive fibrosis, and finally device failure due to the fibrous encapsulation of the implant [59]. Macrophages are the key effector cells in implant-based immunological responses. Once activated, they produce and secrete a vast range of bioactive molecules (e.g., cytokines, chemokines, growth factors, etc.) that modulate and determine the nature and evolution of the inflammatory response through a complex network of interactions [60]. Since the severity of the inflammatory response is closely correlated with the expression of the produced inflammatory mediators [61], the secretion levels of several cytokines, namely TNF-α, IL-1β, and IL-10, were quantified after 2 days of culture. As seen from the graphic (Figure 6a), the macrophages grown onto the smooth PDMS secreted into the culture medium slightly increased levels of TNF-α, in comparison to the analysed micropatterned PDMS surfaces. However, significant differences could also be observed between the microtopographically altered substrates, where the levels of TNF-α released by the cells found on the OLP, OILP, and PLP supports were slightly down-regulated. Furthermore, IL-1β (Figure 6b) exhibited a similar pattern of expression with regard to the OLP, OILP, and PLP substrates, but in this case, the flat PDMS led to a significant reduction in protein expression when compared to some modified surfaces, e.g., CLP (*p* < 0.01). The higher secretion profile trend of IL-1β, synthesised by the cells grown onto the microtopographically altered PDMS surfaces compared to the smooth substrate, could be explained by the macrophages’ preference to migrate to rougher topographies, a phenomenon known in the literature as rugophilia [62]. Moreover, even if the activity of the pro-inflammatory cytokines is perceived in the literature as damaging to the wound healing process, an up-regulation of several cytokines, such as TNF-α, IL-1β, IL-6, IL-18, etc., is absolutely mandatory in the first 24 h after injury/implantation [63], and different studies have demonstrated that an up-regulation in the TNF-α [64] and IL-1β [65] protein levels resulted in an increase in the cellular proliferation and differentiation processes, and in the promotion of an adequate healing process. As opposed to the pro-inflammatory cytokines, the expression secretion levels of IL-10 in the culture medium by the cells grown onto the CLP, SPSP, and CSLP surfaces were significantly down-regulated in comparison to other textured PDMS surfaces and those similar to that of the flat PDMS substrate (Figure 6c).

In addition to cytokine quantification, NO production was assessed by measuring the nitrite concentration accumulated in the culture medium after 48 h. As seen in Figure 6d, the highest levels of NO were recorded by the macrophages grown on the smooth and CLP substrates, while the lowest levels, with an almost three-fold reduction, were observed for the cells grown onto the surface of the PNP support (*p* < 0.0001). Generally, it was observed that under normal physiological conditions, NO exhibits anti-inflammatory effects, but in pathophysiological conditions, such as the introduction of a biomaterial into the host’s organism, the molecule is viewed as a pro-inflammatory mediator, which contributes further to the exacerbation of the inflammatory response [66]. Therefore, in an intense inflammatory microenvironment, where pro-inflammatory cytokine levels are highly up-regulated, the production of NO will also become elevated [66], a phenomenon also observed in our study.

#### 3.2.5. Foreign Body Giant Cells Formation

To further investigate the influence of the developed PDMS surfaces on the inflammatory activity of the RAW 264.7 cells, their potential to induce the formation of FBGCs was evaluated at 7 days post seeding, under pro-inflammatory conditions. FBGCs are a signature component of the biomaterial-induced FBR that was once activated via specific cytokines and can adhere to the surface of the implant and give rise to a fibrotic barrier which isolates the device from the surrounding tissue, leading in time to implant deterioration, loss of function, and in the end, its failure [67]. Increasing knowledge on the hosts’ response to synthetic biomaterials, clearly highlights the potential of different implantable devices to regulate the activity of the immune cells, and implicitly the nature of FBR, by providing microenvironmental cues through diverse surface characteristics including topography [67].

**Figure 7:**
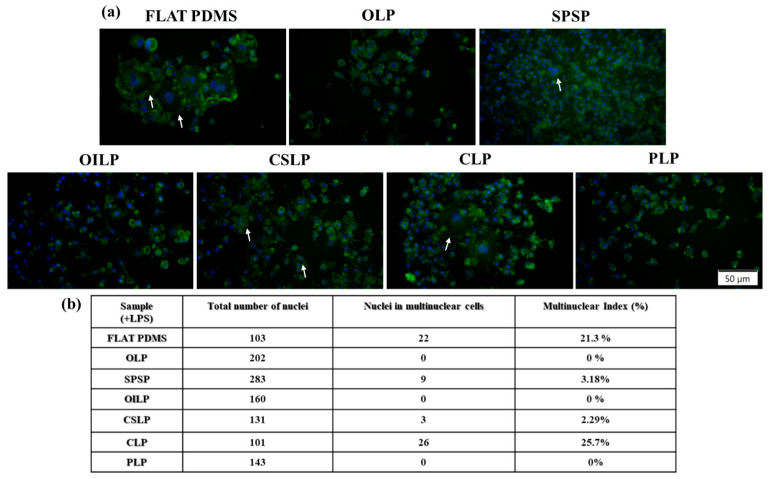
(**a**) Fluorescent images of the FBGCs (white arrows) generated onto the analysed surfaces under LPS stimulation (green fluorescence—actin cytoskeleton, blue fluorescence—nuclei). Scale bar represents 50 µm; (**b**) The “multinuclear index” calculated by examining 10 microscopical fields for each sample.

As seen from Figure 7a, the macrophages grown onto the flat PDMS displayed morphological features specific to multinucleated giant cells, adopting enlarged bodies with numerous filopodial extensions and multiple nuclei (white arrows), whereas on the microtopographically altered PDMS surfaces, the formed FBGCs were smaller and presented a lower number of nuclei (three or four nuclei per cell). Furthermore, even though the cells grown on the OILP, OLP, and PNP surfaces exhibited a morphology specific to the LPS-stimulated cells, no multinucleated giant cells were found, an observation which may suggest that these microtopographies are capable of hindering the macrophage fusion process. Moreover, this hypothesis is also supported by the secretion levels of the analysed inflammatory mediators, where in the case of the aforementioned substrates, an anti-inflammatory trend was noticed. In addition, with the exception of the CLP support (25.7%), the cells cultured onto the rest of the tested microtextured surfaces showed a significant reduction in the multinucleated FBGCs formation incidence (Figure 7b), indicating that in a pro-inflammatory environment, surface microtexturing could dampen macrophage fusion in comparison to smoother surfaces.

## 4. Conclusions

In this study, new PDMS-textured surfaces, based on linear step-like features with various multiscaled regular and irregular arrangements and 250 nm step-like types, were obtained by replication, and their ability to modulate and supress macrophages’ inflammatory activity and bacterial adhesion, respectively, has been investigated in comparison to the flat PDMS surface.

The textured surfaces were characterised by decreased roughness (Sa below 5 μm), with the exception of the gradient circular pattern (Sa = 10.77 μm), and hydrophilic characteristics after UV Ozone treatment. Moreover, the analysed surfaces mainly revealed peaks with flat profiles as well as valleys, with positive skewness and kurtosis values, except for OLP, due to the features arrangement leading to a valley-type profile.

The in vitro results demonstrated that by employing an interrupted microline topography on their surface, the PDMS supports were capable of modulating both the activity of bacteria, as well as the macrophages’ activity, eliciting a less severe biofilm formation and inflammatory response when compared to the smooth surface. Thus, the microtopographically altered PDMS surfaces induced a favourable response in terms of cell survival/proliferation, morphological behaviour, and suppression of macrophage fusion into FBGCs, especially for OILP, OLP, and PLP structures, suggesting the importance of using microtopographies in the process of hindering bacteria adhesion and the macrophage fusion process.

## Figures and Tables

**Table 1:** The parameters describing the replicated surface characteristics.

No.	Arithmetical Mean Height S_a_ (µm)	Root Mean Square Height S_q_ (µm)	Peak to Valley Height S_t_ (µm)	Skewness Parameters Rsk	Kurtosis Parameters Sk
OLP	1.20	1.45	9.49	−0.39	2.58
SPSP	1.01	1.25	8.47	0.16	2.32
OILP	0.47	0.56	4.12	0.343	2.22
CSLP	4.27	4.92	20.15	0.233	1.858
CLP	10.77	12.57	49.3	0.128	1.935
PLP	2.07	2.4	13.8	0.15	1.913

## Data Availability

Data are contained within the article and Supplementary Materials.

## Supplementary Materials

The following supporting information can be downloaded at: https://www.mdpi.com/article/10.3390/polym16213046/s1, Figure S1: SEM image if the moulds and the corresponding replica confirming the maintain of the aspect ratio.

## References

[1] Chang E.I., Hammond D.C. (2018). Clinical Results on Innovation in Breast Implant Design. Plast. Reconstr. Surg..

[2] Deva A.K., Cuss A., Magnusson M., Cooter R. (2019). The “Game of Implants”: A Perspective on the Crisis-Prone History of Breast Implants. Aesthet. Surg. J..

[3] American Society of Plastic Surgeons (2020). Plastic Surgery Statistics Report.

[4] Miranda I., Souza A., Sousa P., Ribeiro J., Castanheira E.M.S., Lima R., Minas G. (2021). Properties and Applications of PDMS for Biomedical Engineering: A Review. J. Funct. Biomater..

[5] Erenay B., Sağlam A.S.Y., Garipcan B., Jandt K.D., Odabaş S. (2022). Bone Surface Mimicked PDMS Membranes Stimulate Osteoblasts and Calcification of Bone Matrix. Biomater. Adv..

[6] Babaei M., Nasernejad B., Sharifikolouei E., Shokrgozar M.A., Bonakdar S. (2022). Bioactivation of 3D Cell-Imprinted Polydimethylsiloxane Surfaces by Bone Protein Nanocoating for Bone Tissue Engineering. ACS Omega.

[7] Maparu A.K., Singh P., Rai B., Sharma A., Sivakumar S. (2024). PDMS Nanoparticles-Decorated PDMS Substrate Promotes Adhesion, Proliferation and Differentiation of Skin Cells. J. Colloid. Interface Sci..

[8] Slepičková Kasálková N., Juřicová V., Fajstavr D., Frýdlová B., Rimpelová S., Švorčík V., Slepička P. (2024). Plasma-Activated Polydimethylsiloxane Microstructured Pattern with Collagen for Improved Myoblast Cell Guidance. Int. J. Mol. Sci..

[9] Varshney N., Sahi A.K., Vajanthri K.Y., Poddar S., Balavigneswaran C.K., Prabhakar A., Rao V., Mahto S.K. (2019). Culturing Melanocytes and Fibroblasts within Three-Dimensional Macroporous PDMS Scaffolds: Towards Skin Dressing Material. Cytotechnology.

[10] Dardouri M., Bettencourt A., Martin V., Carvalho F.A., Colaço B., Gama A., Ramstedt M., Santos N.C., Fernandes M.H., Gomes P.S. (2022). Assuring the Biofunctionalization of Silicone Covalently Bonded to Rhamnolipids: Antibiofilm Activity and Biocompatibility. Pharmaceutics.

[11] Chen C., Chen Y., Lan Y., Tian M., Zhang Y., Lei Z., Fan D. (2023). Effects of Substrate Topography on the Regulation of Human Fibroblasts and Capsule Formation via Modulating Macrophage Polarization. Colloids Surf. B Biointerfaces.

[12] Qin X.H., Senturk B., Valentin J., Malheiro V., Fortunato G., Ren Q., Rottmar M., Maniura-Weber K. (2019). Cell-Membrane-Inspired Silicone Interfaces That Mitigate Proinflammatory Macrophage Activation and Bacterial Adhesion. Langmuir.

[13] Singha P., Locklin J., Handa H. (2017). A Review of the Recent Advances in Antimicrobial Coatings for Urinary Catheters. Acta Biomater..

[14] Armugam A., Teong S.P., Lim D.S.W., Chan S.P., Yi G., Yew D.S., Beh C.W., Zhang Y. (2021). Broad Spectrum Antimicrobial PDMS-Based Biomaterial for Catheter Fabrication. Biomater. Res..

[15] Tohfafarosh M., Sevit A., Patel J., Kiel J.W., Greenspon A., Prutkin J.M., Kurtz S.M. (2016). Characterization of Outer Insulation in Long-Term-Implanted Leads. J. Long. Term. Eff. Med. Implant..

[16] Zare M., Ghomi E.R., Venkatraman P.D., Ramakrishna S. (2021). Silicone-Based Biomaterials for Biomedical Applications: Antimicrobial Strategies and 3D Printing Technologies. J. Appl. Polym. Sci..

[17] Bachour Y. (2021). Capsular Contracture in Breast Implant Surgery: Where Are We Now and Where Are We Going?. Aesthetic Plast. Surg..

[18] Noskovicova N., Hinz B., Pakshir P. (2021). Implant Fibrosis and the Underappreciated Role of Myofibroblasts in the Foreign Body Reaction. Cells.

[19] Chao A.H., Garza R., Povoski S.P. (2016). A Review of the Use of Silicone Implants in Breast Surgery. Expert. Rev. Med. Devices.

[20] Headon H., Kasem A., Mokbel K. (2015). Capsular Contracture after Breast Augmentation: An Update for Clinical Practice. Arch. Plast. Surg..

[21] Atlan M., Nuti G., Wang H., Decker S., Perry T.A. (2018). Breast Implant Surface Texture Impacts Host Tissue Response. J. Mech. Behav. Biomed. Mater..

[22] Yoo B.Y., Kim B.H., Lee J.S., Shin B.H., Kwon H., Koh W.G., Heo C.Y. (2018). Dual Surface Modification of PDMS-Based Silicone Implants to Suppress Capsular Contracture. Acta Biomater..

[23] Choi J., Shin B.H., Kim T., Lee J.S., Kim S., Bin Choy Y., Heo C.Y., Koh W.-G. (2022). Micro-Textured Silicone-Based Implant Fabrication Using Electrospun Fibers as a Sacrificial Template to Suppress Fibrous Capsule Formation. Mater. Sci. Eng. C Mater. Biol. Appl..

[24] Shapouri-Moghaddam A., Mohammadian S., Vazini H., Taghadosi M., Esmaeili S.A., Mardani F., Seifi B., Mohammadi A., Afshari J.T., Sahebkar A. (2018). Macrophage Plasticity, Polarization, and Function in Health and Disease. J. Cell. Physiol..

[25] Lee J.H., Ryu J.Y., Lee J.S., Choi K.Y., Chung H.Y., Cho B.C., Kim K., Lee Y.J., Jin H.K., Bae J.S. (2022). Effect of Breast Silicone Implant Topography on Bacterial Attachment and Growth: An In Vitro Study. Vivo.

[26] Negrescu A.M., Mitran V., Draghicescu W., Popescu S., Pirvu C., Ionascu I., Soare T., Uzun S., Croitoru S.M., Cimpean A. (2022). TiO_2_ Nanotubes Functionalized with Icariin for an Attenuated In Vitro Immune Response and Improved In Vivo Osseointegration. J. Funct. Biomater..

[27] Negrescu A.M., Necula M.G., Gebaur A., Golgovici F., Nica C., Curti F., Iovu H., Costache M., Cimpean A. (2021). In Vitro Macrophage Immunomodulation by Poly(ε-Caprolactone) Based-Coated AZ31 Mg Alloy. Int. J. Mol. Sci..

[28] Walker J.N., Hanson B.M., Pinkner C.L., Simar S.R., Pinkner J.S., Parikh R., Clemens M.W., Hultgren S.J., Myckatyn T.M. (2019). Insights into the Microbiome of Breast Implants and Periprosthetic Tissue in Breast Implant-Associated Anaplastic Large Cell Lymphoma. Sci. Rep..

[29] Gokaltun A., Yarmush M.L., Asatekin A., Usta O.B. (2017). Recent Advances in Nonbiofouling PDMS Surface Modification Strategies Applicable to Microfluidic Technology. Technol. (Singap World Sci.).

[30] Tan S.H., Nguyen N.T., Chua Y.C., Kang T.G. (2010). Oxygen Plasma Treatment for Reducing Hydrophobicity of a Sealed Polydimethylsiloxane Microchannel. Biomicrofluidics.

[31] Nakano H., Kakinoki S., Iwasaki Y. (2021). Long-Lasting Hydrophilic Surface Generated on Poly(Dimethyl Siloxane) with Photoreactive Zwitterionic Polymers. Colloids Surf. B Biointerfaces.

[32] Nistorescu S., Icriverzi M., Florian P., Bonciu A., Marascu V., Dumitrescu N., Pircalabioru G.G., Rusen L., Mocanu A., Roseanu A. (2023). Mitigation of Cellular and Bacterial Adhesion on Laser Modified Poly (2-Methacryloyloxyethyl Phosphorylcholine)/Polydimethylsiloxane Surface. Nanomaterials.

[33] Epstein A.K., Wong T.S., Belisle R.A., Boggs E.M., Aizenberg J. (2012). Liquid-Infused Structured Surfaces with Exceptional Anti-Biofouling Performance. Proc. Natl. Acad. Sci. USA.

[34] Chien H.W., Chen X.Y., Tsai W.P., Lee M. (2020). Inhibition of Biofilm Formation by Rough Shark Skin-Patterned Surfaces. Colloids Surf. B Biointerfaces.

[35] Cheng Y., Feng G., Moraru C.I. (2019). Micro-and Nanotopography Sensitive Bacterial Attachment Mechanisms: A Review. Front. Microbiol..

[36] Linklater D.P., Nguyen H.K.D., Bhadra C.M., Juodkazis S., Ivanova E.P. (2017). Influence of Nanoscale Topology on Bactericidal Efficiency of Black Silicon Surfaces. Nanotechnology.

[37] Helbig R., Günther D., Friedrichs J., Rößler F., Lasagni A., Werner C. (2016). The Impact of Structure Dimensions on Initial Bacterial Adhesion. Biomater. Sci..

[38] Yang M., Ding Y., Ge X., Leng Y. (2015). Control of Bacterial Adhesion and Growth on Honeycomb-like Patterned Surfaces. Colloids Surf. B Biointerfaces.

[39] Xu L.C., Siedlecki C.A. (2017). Protein Adsorption, Platelet Adhesion, and Bacterial Adhesion to Polyethylene-Glycol-Textured Polyurethane Biomaterial Surfaces. J. Biomed. Mater. Res. B Appl. Biomater..

[40] Schwibbert K., Menzel F., Epperlein N., Bonse J., Krüger J. (2019). Bacterial Adhesion on Femtosecond Laser-Modified Polyethylene. Materials.

[41] Tang M., Chen C., Zhu J., Allcock H.R., Siedlecki C.A., Xu L.C. (2021). Inhibition of Bacterial Adhesion and Biofilm Formation by a Textured Fluorinated Alkoxyphosphazene Surface. Bioact. Mater..

[42] Bhushan B., Jung Y.C., Niemietz A., Koch K. (2009). Lotus-like Biomimetic Hierarchical Structures Developed by the Self-Assembly of Tubular Plant Waxes. Langmuir.

[43] Ivanova E.P., Hasan J., Webb H.K., Truong V.K., Watson G.S., Watson J.A., Baulin V.A., Pogodin S., Wang J.Y., Tobin M.J. (2012). Natural Bactericidal Surfaces: Mechanical Rupture of Pseudomonas Aeruginosa Cells by Cicada Wings. Small.

[44] Watson G.S., Green D.W., Schwarzkopf L., Li X., Cribb B.W., Myhra S., Watson J.A. (2015). A Gecko Skin Micro/Nano Structure—A Low Adhesion, Superhydrophobic, Anti-Wetting, Self-Cleaning, Biocompatible, Antibacterial Surface. Acta Biomater..

[45] Li X., Cheung G.S., Watson G.S., Watson J.A., Lin S., Schwarzkopf L., Green D.W. (2016). The Nanotipped Hairs of Gecko Skin and Biotemplated Replicas Impair and/or Kill Pathogenic Bacteria with High Efficiency. Nanoscale.

[46] Bandara C.D., Singh S., Afara I.O., Wolff A., Tesfamichael T., Ostrikov K., Oloyede A. (2017). Bactericidal Effects of Natural Nanotopography of Dragonfly Wing on Escherichia Coli. ACS Appl. Mater. Interfaces.

[47] Ventre M., Natale C.F., Rianna C., Netti P.A. (2014). Topographic Cell Instructive Patterns to Control Cell Adhesion, Polarization and Migration. J. R. Soc. Interface.

[48] Vadiveloo P.K., Keramidaris E., Morrison W.A., Stewart A.G. (2001). Lipopolysaccharide-Induced Cell Cycle Arrest in Macrophages Occurs Independently of Nitric Oxide Synthase II Induction. Biochim. Biophys. Acta.

[49] Wolfenson H., Yang B., Sheetz M.P. (2019). Steps in Mechanotransduction Pathways That Control Cell Morphology. Annu. Rev. Physiol..

[50] Leclech C., Villard C. (2020). Cellular and Subcellular Contact Guidance on Microfabricated Substrates. Front. Bioeng. Biotechnol..

[51] Nguyen A.T., Sathe S.R., Yim E.K.F. (2016). From Nano to Micro: Topographical Scale and Its Impact on Cell Adhesion, Morphology and Contact Guidance. J. Phys. Condens. Matter.

[52] Jeon H., Koo S., Reese W.M., Loskill P., Grigoropoulos C.P., Healy K.E. (2015). Directing Cell Migration and Organization via Nanocrater-Patterned Cell-Repellent Interfaces. Nat. Mater..

[53] Tawfick S., De Volder M., Copic D., Park S.J., Oliver C.R., Polsen E.S., Roberts M.J., Hart A.J. (2012). Engineering of Micro and Nanostructured Surfaces with Anisotropic Geometries and Properties. Adv. Mater..

[54] Nikkhah M., Edalat F., Manoucheri S., Khademhosseini A. (2012). Engineering Microscale Topographies to Control the Cell-Substrate Interface. Biomaterials.

[55] McWhorter F.Y., Davis C.T., Liu W.F. (2015). Physical and Mechanical Regulation of Macrophage Phenotype and Function. Cell Mol. Life Sci..

[56] Saltel F., Chabadel A., Bonnelye E., Jurdic P. (2008). Actin Cytoskeletal Organisation in Osteoclasts: A Model to Decipher Transmigration and Matrix Degradation. Eur. J. Cell Biol..

[57] Chen S., Jones J.A., Xu Y., Low H.Y., Anderson J.M., Leong K.W. (2010). Characterization of Topographical Effects on Macrophage Behavior in a Foreign Body Response Model. Biomaterials.

[58] Bettinger C.J., Orrick B., Misra A., Langer R., Borenstein J.T. (2006). Microfabrication of Poly (Glycerol-Sebacate) for Contact Guidance Applications. Biomaterials.

[59] Anderson J.M., Rodriguez A., Chang D.T. (2008). Foreign Body Reaction to Biomaterials. Semin. Immunol..

[60] Commins S.P., Borish L., Steinke J.W. (2010). Immunologic Messenger Molecules: Cytokines, Interferons, and Chemokines. J. Allergy Clin. Immunol..

[61] Chandorkar Y., Ravikumar K., Basu B. (2019). The Foreign Body Response Demystified. ACS Biomater. Sci. Eng..

[62] Rich A., Harris A.K. (1981). Anomalous Preferences of Cultured Macrophages for Hydrophobic and Roughened Substrata. J. Cell Sci..

[63] Rundle C.H., Wang H., Yu H., Chadwick R.B., Davis E.I., Wergedal J.E., Lau K.H.W., Mohan S., Ryaby J.T., Baylink D.J. (2006). Microarray Analysis of Gene Expression during the Inflammation and Endochondral Bone Formation Stages of Rat Femur Fracture Repair. Bone.

[64] Glass G.E., Chan J.K., Freidin A., Feldmann M., Horwood N.J., Nanchahal J. (2011). TNF-α Promotes Fracture Repair by Augmenting the Recruitment and Differentiation of Muscle-Derived Stromal Cells. Proc. Natl. Acad. Sci. USA.

[65] Lange J., Sapozhnikova A., Lu C., Hu D., Li X., Miclau T., Marcucio R.S. (2010). Action of IL-1β during Fracture Healing. J. Orthop. Res..

[66] Sharma J.N., Al-Omran A., Parvathy S.S. (2007). Role of Nitric Oxide in Inflammatory Diseases. Inflammopharmacology.

[67] Mariani E., Lisignoli G., Borzì R.M., Pulsatelli L. (2019). Biomaterials: Foreign Bodies or Tuners for the Immune Response?. Int. J. Mol. Sci..

