# Supplementary material for: Macrophage Immunomodulation and Suppression of Bacterial Growth by Polydimethylsiloxane Surface-Interrupted Microlines’ Topography Targeting Breast Implant Applications
Source: Polymers (Basel). 2024 Oct 29;16(21):3046. doi: 10.3390/polym16213046 (PMC11548769; doi:10.3390/polym16213046)

Supplementary Materials

Figure S1. SEM image if the moulds and the corresponding replica confirming the maintain of the aspect ratio.

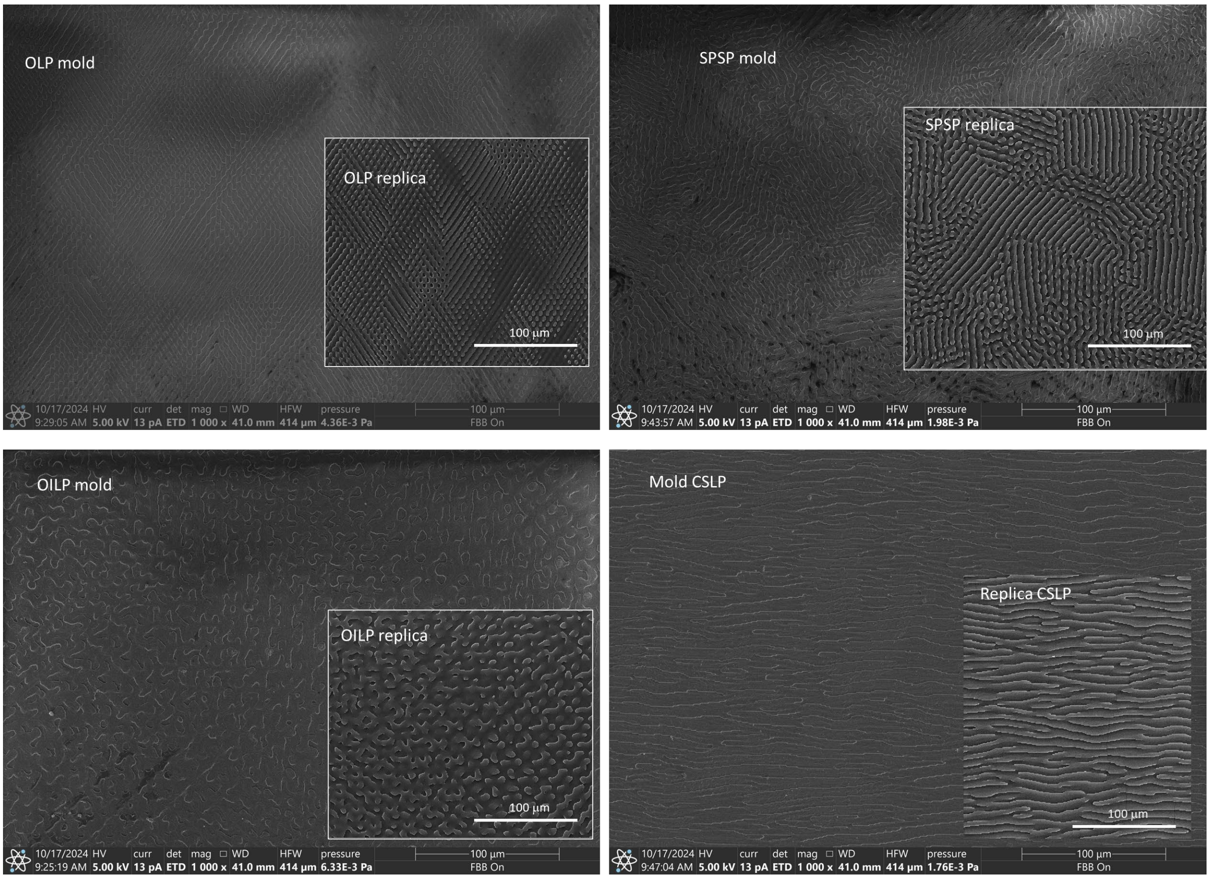

Supplement: Supplementary file 1 [file polymers-16-03046-s001.zip › polymers-3267480-supplementary.pdf]
